# Impact of the COVID-19 Pandemic on Chronic Liver Disease and Cirrhosis Mortality in the United States: A Divergent Trend Analysis by Underlying Etiology (2018-2024)

**DOI:** 10.7759/cureus.116773

**Published:** 2026-09-23

**Authors:** Elizabeth Blanco Espinosa, Yusvell Mori Gonzalez, Leandro Patterson, Javier A Mendoza, Wendy Larrondo del Campo, Gabriela Sanchez, Francisco Sotolongo Castro, Yurisleydis Dominguez, Juan T Vargas Rivera, Aitor J Roche Perez, Carlos Acosta-Batista

**Affiliations:** 1 General Practice, Ceda Orthopedic Group, Miami, USA; 2 Clinical Research, Primary Care - Research Initiative (PCRI), Miami Lakes, USA; 3 Primary Care, Hermes Medical Group, Miami, USA; 4 Internal Medicine, Mount Sinai Medical Center, Hialeah, USA; 5 General Practice, American Medical Association, Hialeah, USA; 6 Urology, Mission City Urology, San Antonio, USA; 7 Research and Development, Tricocenter Miami, Miami Lakes, USA

**Keywords:** alcohol-related liver disease, cdc wonder, chronic liver disease, cirrhosis, covid-19 pandemic, health disparities, mortality, urbanization, years of potential life lost

## Abstract

Background: Chronic liver disease (CLD) and cirrhosis are major causes of mortality in the United States, with substantial variation by etiology, demographics, and geography. Alcohol-related liver disease (ALD) mortality increased before the COVID-19 pandemic and accelerated during its early phase. Whether alcohol-related and non-alcohol-related CLD and cirrhosis followed divergent mortality trajectories during and after the pandemic remains incompletely characterized.

Methods: We conducted a population-based mortality study using the Centers for Disease Control and Prevention Wide-ranging Online Data for Epidemiologic Research (CDC WONDER) National Vital Statistics System data for deaths occurring from 2018 to 2024. Deaths were classified as ALD (K70) or CLD and cirrhosis not coded as alcohol-related (K73-K74). Periods were defined as pre-pandemic (2018-2019), pandemic (2020-2021), and post-acute pandemic (2022-2024). Outcomes included mortality counts, age-adjusted mortality rates, percentage changes, and years of potential life lost before age 75 (YPLL-75). All data aggregation, rate calculations, and statistical analyses, including confidence interval derivations, were performed using Python Version 3.10 (Python Software Foundation, Wilmington, Delaware, United States), utilizing the pandas and NumPy libraries. Analyses were stratified by sex, race and ethnicity, urbanization, and state.

Results: K70 deaths increased from 47,277 pre-pandemic to 62,601 during 2020-2021 (+32.4%), compared with an increase in K73-K74 deaths from 39,895 to 45,613 (+14.3%). During 2022-2024, K70 accounted for 87,294 deaths versus 71,982 for K73-K74. Average annual YPLL-75 increased by 38.8% for K70 compared with 19.5% for K73-K74, with YPLL per death consistently higher for K70. Marked disparities were observed by sex, race, urbanization, and state. American Indian or Alaska Native populations had the highest K70 mortality rates and largest pandemic-era increase, while nonmetropolitan populations experienced greater increases than metropolitan populations.

Conclusions: Alcohol-related and non-alcohol-related CLD and cirrhosis demonstrated distinct mortality trajectories during the pandemic era, with ALD contributing to a disproportionate burden of premature mortality. These findings describe temporal and etiologic differences in mortality and may inform targeted prevention and treatment strategies, although the observational design does not permit causal attribution to specific pandemic-related mechanisms.

## Introduction

Chronic liver disease (CLD) and cirrhosis represent major causes of morbidity and mortality in the United States and encompass a heterogeneous spectrum of conditions, including alcohol-related liver disease (ALD), chronic viral hepatitis, metabolic liver disease, hepatic fibrosis, and cirrhosis. Mortality from CLD has changed substantially over the past several decades, with important differences according to underlying etiology, age, sex, race and ethnicity, and geographic location [1-4].

ALD has emerged as an increasingly important contributor to this mortality burden. National mortality studies have documented increasing ALD mortality over the past several decades, particularly among younger adults and women. Tapper and Parikh reported a marked increase in cirrhosis mortality among adults aged 25-34 years between 2009 and 2016, with the increase in this age group driven entirely by ALD [1]. Subsequent studies demonstrated continued increases in etiology-specific liver disease mortality, including substantial increases in ALD [2-4].

The COVID-19 pandemic occurred against this background of increasing ALD and CLD mortality and was associated with multiple potential sources of disruption, including changes in alcohol consumption, social isolation, psychological stress, healthcare access, treatment interruptions, and reduced utilization of preventive and specialty services. Modeling studies predicted that increased alcohol consumption during the pandemic could substantially increase the future burden of ALD [5].

Consistent with these concerns, several national studies demonstrated marked increases in liver disease mortality during the pandemic. Kim et al. reported an acceleration in CLD and cirrhosis mortality during 2020, particularly among younger adults [6]. Subsequent etiology-specific analyses demonstrated particularly pronounced increases in ALD mortality during the pandemic. Kim et al. reported a substantial acceleration in ALD mortality during 2020, while Gao et al. found that ALD mortality increased markedly above pre-pandemic trends, particularly among younger adults and American Indian or Alaska Native populations [7,8].

Paik et al. subsequently evaluated CLD mortality through 2021 using National Vital Statistics System data and demonstrated substantial acceleration in cirrhosis-specific mortality during 2019-2021. Their analysis also showed particularly large increases in ALD-related mortality among American Indian or Alaska Native populations and selected female racial groups [9].

These findings establish that the COVID-19 pandemic was associated with important changes in liver disease mortality. However, an important epidemiologic question remains: were these changes uniform across liver disease etiologies, or did ALD and CLD and cirrhosis not coded as alcohol-related follow divergent mortality trajectories?

This distinction is clinically and epidemiologically relevant. ALD may be particularly sensitive to pandemic-associated changes in alcohol consumption, behavioral health, social isolation, and access to addiction treatment. However, these potential mechanisms cannot be directly evaluated using population-level mortality data. In contrast, mortality from other CLD and cirrhosis may have been influenced by interruptions in chronic disease management, delayed diagnosis, reduced surveillance, and disruption of specialty care. These factors represent potential explanations rather than mechanisms that can be established by the present study. Several studies have addressed individual components of this question but have used different analytic frameworks. Kim et al. and other national mortality studies have evaluated CLD and cirrhosis using the International Classification of Diseases, Tenth Revision (ICD-10)-based definitions and identified ALD using multiple-cause-of-death definitions, allowing comparison among ALD, nonalcoholic fatty liver disease (NAFLD), viral hepatitis, and other etiologies but not a direct comparison of K70 versus K73-K74 as mutually distinct underlying-cause categories [7-9].

Race and ethnicity represent another important dimension of liver disease mortality disparities. Previous analyses have demonstrated substantial racial and ethnic differences in ALD and overall liver disease mortality, including particularly high mortality among American Indian or Alaska Native populations [8,10-12]. However, these studies have generally evaluated racial differences within individual disease categories rather than determining whether the difference between ALD and CLD and cirrhosis not coded as alcohol-related mortality varies across racial and ethnic groups. Recent studies have also expanded the temporal coverage of ALD mortality. Pan et al. analyzed ALD mortality from 1999 through 2022 using ICD-10 K70 and demonstrated substantial acceleration beginning in 2018, with particularly pronounced increases among women, younger adults, and American Indian or Alaska Native populations [13]. Yu et al. subsequently extended ALD mortality surveillance from 1999 through 2024 using the Centers for Disease Control and Prevention Wide-ranging Online Data for Epidemiologic Research (CDC WONDER) data and reported a marked increase from 2018 through 2021 followed by partial attenuation thereafter [14].

These recent findings are important because ALD mortality through 2024 has now been characterized independently. Therefore, the contribution of the present study is not simply to describe the increase in ALD mortality during COVID-19 or to extend ALD surveillance through 2024. Rather, the central question is whether the trajectory of ALD differs from that of CLD and cirrhosis not coded as alcohol-related when both are evaluated within the same national population and calendar period.

Knowledge gap

To our knowledge, few national mortality studies have directly compared K70 versus K73-K74 as separate underlying-cause categories across the transition from the pre-pandemic period through the COVID-19 pandemic and into the post-acute pandemic period through 2024. Furthermore, previous studies have not simultaneously evaluated whether this etiologic divergence differs across demographic and geographic populations while also quantifying its contribution to premature mortality using years of potential life lost (YPLL). The present study therefore evaluates divergent mortality trajectories between ALD (K70) and CLD and cirrhosis not coded as alcohol-related (K73-K74) from 2018 through 2024.

The primary objective of this study was to compare mortality trajectories for ALD (K70) and CLD and cirrhosis not coded as alcohol-related (K73-K74) across three prespecified periods: pre-pandemic (2018-2019), pandemic (2020-2021), and post-acute pandemic (2022-2024). Secondary objectives were to characterize differences in these mortality patterns across demographic and geographic subgroups and to quantify the associated burden of premature mortality using years of potential life lost before age 75 (YPLL-75). We hypothesized that K70 mortality would demonstrate a greater increase during the pandemic period and a distinct post-acute trajectory compared with K73-K74 mortality.

## Materials and methods

Study design and data source 

We conducted a retrospective population-based cross-sectional study using final mortality data extracted from the CDC WONDER Multiple Cause of Death files (2018-2024), utilizing the Single-Race Population Estimates database. The exact query parameters defined the underlying cause of death as ICD-10 K70 or K73-K74. To calculate multi-year rates for our predefined periods, aggregate death counts for the grouped years were divided by the sum of the population estimates for those corresponding years.

Deaths were categorized into two etiologic groups according to the ICD-10: ALD (K70) and CLD and cirrhosis not coded as alcohol-related (K73-K74). The analysis compared mortality across three prespecified periods: pre-pandemic (2018-2019), pandemic period (2020-2021), and post-acute period (2022-2024).

The study evaluated death counts, mortality rates, temporal changes, premature mortality using YPLL-75, and demographic and geographic variation by sex, race/ethnicity, urbanization, and state. The analysis was descriptive and was designed to characterize observed temporal and geographic patterns rather than to establish causal relationships between the COVID-19 pandemic and liver disease mortality.

Study period

The study period was divided a priori into three periods: the pre-pandemic period (2018-2019), the pandemic period (2020-2021), and the post-acute pandemic period (2022-2024).

The term "post-acute pandemic" was used rather than "post-pandemic" to avoid implying that the COVID-19 pandemic ended at a specific calendar date.

Liver disease definitions

Deaths were classified according to the underlying cause of death. ALD was defined as ICD-10 code K70 (alcoholic liver disease), whereas CLD and cirrhosis not coded as alcohol-related were defined as ICD-10 codes K73-K74, representing chronic hepatitis not elsewhere classified and fibrosis and cirrhosis of the liver. K73-K74 was interpreted as CLD and cirrhosis not coded as alcohol-related on the death certificate rather than definitive evidence that alcohol did not contribute to disease pathogenesis. The two categories were analyzed separately to evaluate divergent mortality trajectories.

Population and demographic variables

The study population included US residents with an underlying cause of death coded as K70 or K73-K74. The following demographic variables were evaluated, including age, sex, single-race classification, Hispanic ethnicity, county-level urbanization, and state of residence.

Race was categorized using the single-race classification available in CDC WONDER mortality data. Hispanic ethnicity was analyzed separately from race according to CDC WONDER classification.

Urbanization

County-level urbanization was evaluated to assess differences between metropolitan and nonmetropolitan populations. Urbanization was categorized using the National Center for Health Statistics urban-rural classification available for the study data. Metropolitan jurisdictions included large central, large fringe, medium, and small metropolitan counties. Nonmetropolitan jurisdictions included micropolitan and noncore counties.

Mortality outcomes

The primary outcomes were average annual death counts and crude mortality rates per 100,000 population. Crude rates were intentionally utilized as the primary metric for all demographic and geographic stratifications to accurately reflect the true absolute burden placed on local healthcare systems and to prevent statistical distortion or excessive cell suppression that frequently occurs when age-adjusting highly stratified, smaller population subsets. Secondary outcomes included the number of deaths, period-specific percentage changes, YPLL-75, YPLL per death, and YPLL rate per 100,000 population.

YPLL

YPLL-75 were used as a measure of premature mortality. For deaths occurring before age 75 years, YPLL was estimated as 75 years minus the midpoint of the corresponding 10-year age category: YPLL = 75 − midpoint of age category. 

Deaths occurring at age 75 years or older contributed zero YPLL.

Total YPLL was calculated as the sum of individual years of potential life lost. 

YPLL was expressed as total YPLL, average annual YPLL, YPLL per death, and YPLL rate per 100,000 population.

YPLL was calculated separately for K70 and K73-K74 and compared across the three study periods.

The standard 10-year age groups provided by CDC WONDER were utilized. The exact mathematical midpoints applied for the YPLL-75 calculation were as follows: <1 year (0.5), 1-4 years (2.5), 5-14 years (9.5), 15-24 years (19.5), 25-34 years (29.5), 35-44 years (39.5), 45-54 years (49.5), 55-64 years (59.5), and 65-74 years (69.5).

Statistical analysis

Mortality data were extracted from the CDC WONDER database. The study evaluated mortality trajectories by defining two underlying-cause cohorts based on ICD-10 codes: ALD (K70) and CLD and cirrhosis not coded as alcohol-related (K73-K74). Temporal trends were evaluated across three predefined study periods: pre-pandemic baseline (2018-2019), pandemic (2020-2021), and post-acute pandemic (2022-2024).

Baseline descriptive statistics included absolute death counts, average annual deaths, and crude mortality rates expressed per 100,000 population. Rather than relying solely on CDC-provided intervals, 95% confidence intervals (CIs) for crude rates were independently calculated utilizing the Poisson distribution standard error methodology to ensure mathematical consistency across all custom multi-year aggregated periods. Relative percentage changes were computed to quantify the magnitude of mortality surges between the pre-pandemic and acute pandemic phases, as well as the subsequent trajectory shifts during the post-acute phase.

To assess the burden of premature death, YPLL-75 were calculated for both etiologies. YPLL was computed by subtracting the midpoint of the decedent's 10-year age cohort from the standard life expectancy benchmark of 75 years; deaths occurring at age 75 or older contributed zero YPLL to the analysis.

Subgroup analyses were conducted to evaluate structural disparities across sex, single-race classifications, county-level urbanization (dichotomized into metropolitan versus nonmetropolitan categories), and state-level jurisdictions. In accordance with CDC WONDER confidentiality constraints, cells representing fewer than 10 deaths were systematically suppressed. To reduce the impact of cell suppression on highly stratified analyses, crude rates for racial disparities were extracted directly from pure demographic files rather than heavily cross-tabulated geographic extractions. Furthermore, for post-acute urbanization rates where updated population denominators were suppressed or unavailable in CDC WONDER, the 2021 intercensal population estimates were used as fixed proxy denominators for the 2022-2024 period. This approach was adopted because updated population denominators for certain recent geographic strata were unavailable or suppressed in the CDC WONDER extraction. The use of the most recent available valid denominator allowed the estimation of crude post-acute mortality rates while preserving the underlying mortality counts. All data aggregation, rate calculations, and statistical analyses, including confidence interval derivations, were performed using Python Version 3.10 (Python Software Foundation, Wilmington, Delaware, United States) utilizing the pandas and NumPy libraries.

## Results

Divergent absolute mortality trajectories

During the study period 2018-2024, temporal analyses revealed a divergence in the absolute mortality trajectories between ALD (K70) and CLD and cirrhosis not coded as alcohol-related (K73-K74) across the three predefined temporal phases.

In the pre-pandemic baseline period (2018-2019), K70 accounted for an average of 23,639 deaths annually, while K73-K74 accounted for an average of 19,948 deaths. During the pandemic period, alcohol-related mortality experienced a substantial increase. The average annual K70 deaths escalated to 31,301, representing a 32.4% relative increase from the pre-pandemic baseline. In contrast, mortality from CLD and cirrhosis not coded as alcohol-related (K73-K74) experienced a comparatively smaller increase of 14.3%, rising to 22,807 average annual deaths.

However, during the post-acute pandemic period (2022-2024), the etiologic trajectories exhibited different patterns. ALD mortality declined by 7% relative to the pandemic-period average, decreasing to an average of 29,098 annual deaths. Conversely, mortality from CLD and cirrhosis not coded as alcohol-related (K73-K74) continued to increase during the post-acute period, rising an additional 5.2% above its 2020-2021 levels to reach 23,994 average annual deaths in the post-acute period (Figure 1).

**Figure 1:**
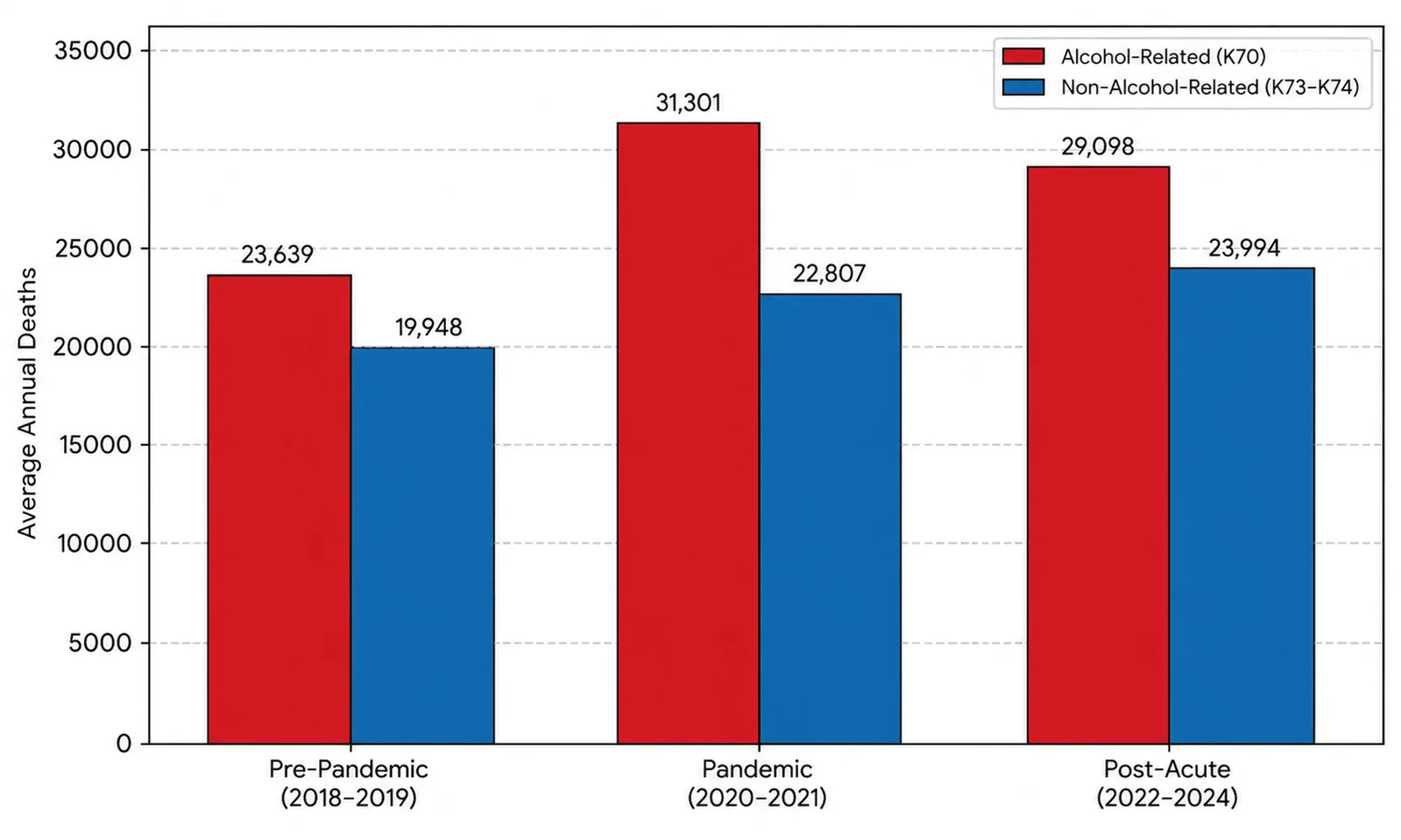
Divergent absolute mortality trajectories by liver disease etiology (2018-2024) Bar chart illustrating the average annual deaths attributed to alcohol-related liver disease (K70; red bars) versus chronic liver disease and cirrhosis not coded as alcohol-related (K73-K74; blue bars) across three predefined temporal phases. The data reveal a distinct "shock and rebound" pattern. Alcohol-related mortality increased by +32.4% from the pre-pandemic to the pandemic period, before attenuating by -7% during the post-acute period. Conversely, mortality from chronic liver disease and cirrhosis not coded as alcohol-related exhibited a delayed increase, with average annual deaths surging by an additional +5.2% in the post-acute era after a comparatively modest initial pandemic increase (+14.3%).

Premature Mortality Burden: YPLL-75

To quantify the impact of these divergent trajectories on premature mortality, YPLL-75 were calculated for both etiologies (Table 1). The YPLL-75 analysis demonstrated that ALD was associated with a substantially greater premature mortality burden than CLD and cirrhosis not coded as alcohol-related. In the pre-pandemic period, K70 accounted for an average of 433,940 YPLL annually, nearly double the burden of K73-K74 (214,373 YPLL).

**Table 1:** Premature mortality burden: YPLL-75 by liver disease etiology (2018-2024) YPLL-75 was calculated by subtracting the midpoint of the decedent's 10-year age cohort from 75. Deaths occurring at age 75 or older contributed zero YPLL. The sum of subcategories may not perfectly match the national macro-total (N=47,282) due to the CDC WONDER privacy policy, which suppresses exact death counts for demographic strata with fewer than 10 events. YPLL: years of potential life lost; YPLL-75: years of potential life lost before age 75; CDC WONDER: Centers for Disease Control and Prevention Wide-ranging Online Data for Epidemiologic Research

Etiology and study period	Total deaths	Total YPLL-75	Average annual YPLL-75	YPLL per death	% change in annual YPLL (vs. prior period)
Alcohol-related (K70)					
Pre-pandemic (2018-2019)	47,277	867,880	433,940	18.4	-
Pandemic (2020-2021)	62,601	1,204,381	602,190	19.2	+38.77%
Post-acute (2022-2024)	87,294	1,637,710	545,903	18.8	-9.35%
Chronic liver disease and cirrhosis not coded as alcohol-related (K73-K74)					
Pre-pandemic (2018-2019)	39,895	428,747	214,373	10.7	-
Pandemic (2020-2021)	45,613	512,278	256,139	11.2	+19.48%
Post-acute (2022-2024)	71,982	752,727	250,909	10.5	-2.04%

During the acute pandemic (2020-2021), the premature mortality burden for ALD increased by +38.77%, reaching an unprecedented 602,190 average annual YPLL. This finding indicates that the increase in K70 mortality was accompanied by a substantial increase in premature mortality burden. During the same acute phase, the YPLL for K73-K74 increased by +19.48% (to 256,139 average annual YPLL). In the post-acute period (2022-2024), the premature mortality burden for both etiologies contracted slightly (-9.34% for K70 and -2.04% for K73-K74), though both remained elevated above their pre-pandemic baselines. Notably, the YPLL-per-death ratio, a metric indicating the relative age distribution of decedents with respect to the 75-year threshold, was consistently higher for ALD. Throughout the entire study window, K70 deaths were associated with an average of 18.3-19.2 years of potential life lost per death, compared with 10.4-11.2 years for K73-K74 deaths.

Sex disparities and the narrowing sex gap

Analysis of crude mortality rates demonstrated a higher absolute mortality rate among males for ALD (K70); however, the proportional trajectory revealed a narrowing sex gap. During the pre-pandemic baseline, the K70 mortality rate for males was 14.6 per 100,000 compared to 6.6 per 100,000 for females (Figure 2A). During the acute pandemic phase, males experienced an increase to 18.6 per 100,000 (+27.7%), while females experienced a larger relative increase of +33.4%, reaching 8.8 deaths per 100,000.

**Figure 2:**
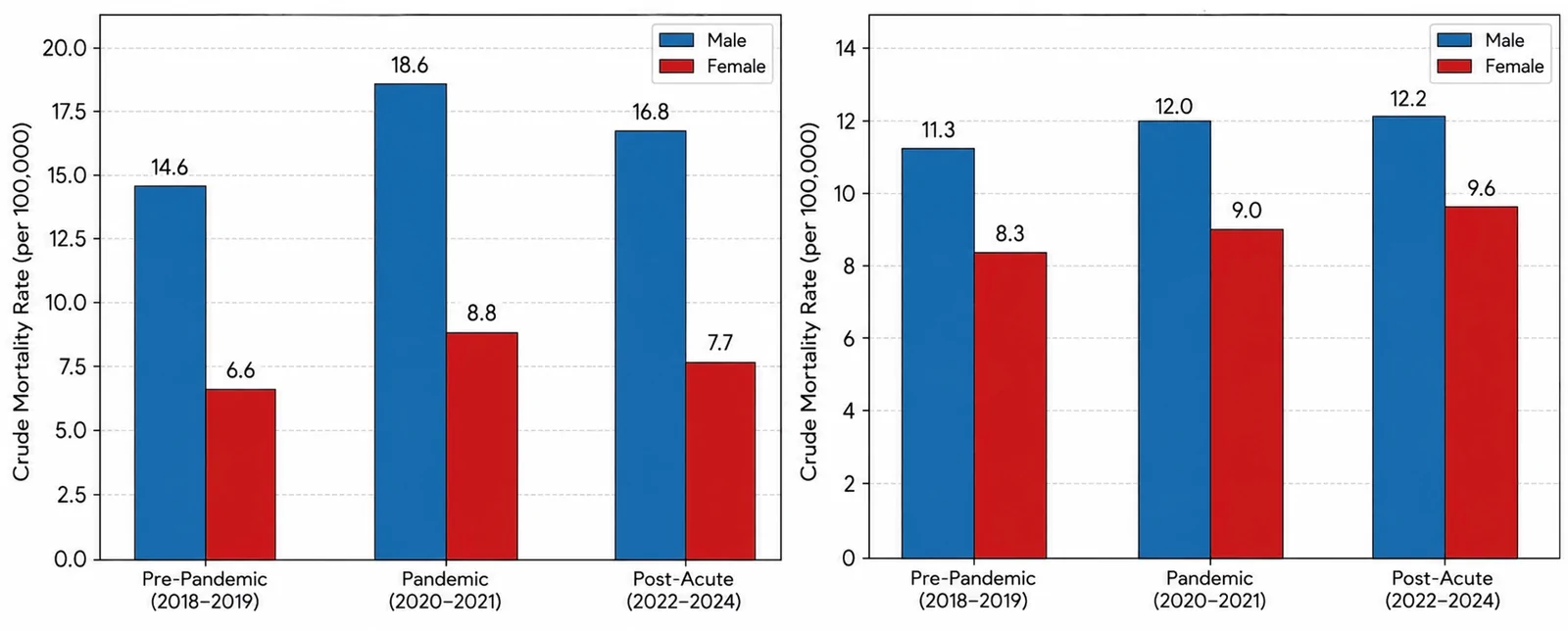
Sex disparities in crude mortality rates by liver disease etiology (2018-2024) Comparative bar charts detailing the crude mortality rates (per 100,000 population) stratified by sex across the pre-pandemic, pandemic, and post-acute periods. (A) Alcohol-related mortality (K70) demonstrates a persistent male predominance in absolute terms; however, females experienced a steeper relative acceleration during the acute pandemic (+33.4% vs. +27.7% for males), indicating a narrowing gender gap in vulnerability. (B) Mortality from chronic liver disease and cirrhosis not coded as alcohol-related (K73-K74) reveals a much narrower baseline gender disparity. Notably, females exhibited continued susceptibility to disruptions in chronic disease management, with mortality rates maintaining a steady climb into the post-acute period (+5.8%), while the male mortality rate plateaued (+1.5%).

A different pattern emerged in CLD and cirrhosis not coded as alcohol-related (K73-K74). The baseline sex disparity was considerably narrower (11.3 for males vs. 8.3 for females). Females exhibited a greater relative increase in K73-K74 mortality during the pandemic (+9.3%) and continued to experience an additional +5.8% increase during the post-acute period. In contrast, the male rate increased by 5.9% during the pandemic and by 1.5% during the post-acute period (Figure 2B).

Racial disparities: the catastrophic impact on American Indian/Alaska Native populations 

Stratification by Single Race classification demonstrated substantial etiology-specific differences in mortality rates and temporal changes across racial groups (Table 2).

**Table 2:** Racial disparities: crude mortality rates by liver disease etiology (2018-2024) Rates are expressed per 100,000 population. Values in parenthesis represent the 95% CI. The data highlight that the massive mortality surge in the American Indian/Alaska Native population was heavily restricted to alcohol-related (K70) disease, escaping the margins of statistical error from the pre-pandemic baseline.

Etiology and race	Pre-pandemic (2018-2019)	Pandemic (2020-2021)	Post-acute (2022-2024)	% change (pre to pan)
Alcohol-related (K70)				
American Indian or Alaska Native	53.4 (50.8-56.0)	76.4 (73.4-79.3)	59.7 (57.6-61.8)	+43%
Asian	2.6 (2.4-2.8)	3.4 (3.2-3.7)	3.3 (3.1-3.5)	+32.4%
Black or African American	6.8 (6.6-7.0)	8.9 (8.7-9.2)	7.4 (7.2-7.5)	+30.9%
White	11.3 (11.2-11.4)	14.6 (14.5-14.8)	13.1 (13.0-13.2)	+29.5%
Chronic liver disease and cirrhosis not coded as alcohol-related (K73-K74)				
American Indian or Alaska Native	22.3 (20.4-24.2)	22.8 (21.1-24.5)	23.4 (22.1-24.8)	+2.4%
Asian	5.1 (4.7-5.4)	4.9 (4.6-5.2)	5.3 (5.0-5.6)	-3.5%
Black or African American	7.7 (7.4-8.0)	6.9 (6.6-7.1)	6.4 (6.2-6.6)	-11.1%
White	10.1 (10.0-10.2)	11.2 (11.1-11.3)	11.7 (11.6-11.8)	+11.1%

During the pre-pandemic period, the K70 mortality rate for the American Indian or Alaska Native population was 53.4 per 100,000. During the acute pandemic phase, this rate increased to 76.4 per 100,000, representing a +43% relative increase. The mortality rate for CLD and cirrhosis not coded as alcohol-related (K73-K74) among the American Indian or Alaska Native population changed by +2.4% during the same transition.

Divergent racial trajectories were also observed when comparing Black and White populations. During the acute pandemic, K70 mortality increased by +30.9% among Black populations and +29.5% among White populations. For K73-K74 mortality, White populations experienced a +11.1% increase, whereas Black populations experienced a -11.1% change during the pandemic period. In the post-acute period (2022-2024), K70 mortality decreased by 17.5% among Black populations and by 10.4% among White populations.

Urbanization disparities: the rural vulnerability to alcohol-related mortality

To evaluate geographic variation in mortality, rates were stratified by county-level urbanization, comparing metropolitan and nonmetropolitan jurisdictions. During the pre-pandemic baseline, nonmetropolitan areas exhibited higher crude mortality rates for both etiologies compared with metropolitan areas (Table 3).

**Table 3:** Urbanization disparities: crude mortality rates by liver disease etiology (2018-2024) Rates are expressed per 100,000 population. Parentheses indicate 95% CI. Metropolitan jurisdictions encompass large central, large fringe, medium, and small metros. Nonmetropolitan jurisdictions encompass micropolitan and noncore (rural) counties.

Etiology and urbanization	Pre-pandemic (2018-2019)	Pandemic (2020-2021)	Post-acute (2022-2024)	Relative change (pre to pan)
Alcohol-related (K70)				
Metropolitan	6.9 (6.9-7.0)	9.0 (8.9-9.1)	8.3 (8.3-8.4)	+29.5%
Nonmetropolitan (rural)	9.0 (8.8-9.2)	12.5 (12.2-12.7)	11.5 (11.4-11.7)	+38.7%
Chronic liver disease and cirrhosis not coded as alcohol-related (K73-K74)				
Metropolitan	5.7 (5.6-5.7)	6.4 (6.3-6.5)	6.7 (6.6-6.7)	+12.6%
Nonmetropolitan (rural)	8.9 (8.7-9.1)	10.3 (10.1-10.5)	11.0 (10.8-11.2)	+15.5%

For ALD (K70), mortality in nonmetropolitan areas increased from 9.0 per 100,000 during the pre-pandemic period to 12.5 per 100,000 during the pandemic, representing a +38.7% relative increase. Metropolitan areas also experienced an increase in K70 mortality, from 6.9 to 9.0 per 100,000 (+29.5%).

For CLD and cirrhosis not coded as alcohol-related (K73-K74), the relative pandemic-period increase was 15.5% in nonmetropolitan areas and 12.6% in metropolitan areas.

State-level heterogeneity

State-level analysis demonstrated substantial geographic variation in the change in liver disease mortality during the pandemic period. Several states showed relative increases in K70 mortality that were substantially larger than the national average. Hawaii and Mississippi had the largest relative increases among the states shown in Table 4, with increases of +355.1% and +195.1%, respectively. Other states with large increases included Alaska (+148.3%) and Delaware (+138.1%).

**Table 4:** State-level extremes: top states with the highest relative surge in alcohol-related mortality (K70) Rates are expressed as crude mortality per 100,000 population (95% CI). Large relative percentage changes should be interpreted cautiously, particularly in states with smaller underlying populations or lower baseline mortality rates. Confidence intervals reflect statistical uncertainty around the estimated rates but do not establish causality.

State	Pre-pandemic (2018-2019)	Pandemic (2020-2021)	Post-acute (2022-2024)	% change (pan vs. pre)	% change (post vs. pan)
Hawaii	3.8 (3.0-4.5)	17.1 (14.9-19.3)	8.2 (6.6-9.9)	+355.1%	-51.9%
Mississippi	4.6 (4.0-5.2)	13.6 (12.6-14.7)	10.9 (10.2-11.6)	+195.1%	-19.6%
Alaska	12.0 (10.2-13.8)	29.8 (27.0-32.6)	20.9 (18.8-23.1)	+148.3%	-29.6%
Delaware	4.5 (3.5-5.5)	10.7 (9.3-12.2)	8.8 (7.6-10.1)	+138.1%	-17.2%
District of Columbia	5.0 (3.9-6.2)	11.0 (9.5-12.6)	9.4 (7.0-11.9)	+118.5%	-14.4%

In contrast, changes in mortality from CLD and cirrhosis not coded as alcohol-related (K73-K74) were comparatively smaller during the acute pandemic period, although several states demonstrated continued increases during the post-acute period (Table 5). The states with the highest pandemic-period increases for K73-K74, such as South Dakota (+56.5%) and Oregon (+55.3%), experienced relative increases that were smaller than those observed among the states with the largest K70 increases.

**Table 5:** State-level extremes: top states with the highest surge in non-alcohol-related mortality (K73-K74) Rates are expressed as crude mortality per 100,000 population (95% CI). Unlike the alcohol-related surges which mostly receded, several top states in this K73-K74 category (e.g., Nebraska, Arkansas, Iowa) demonstrated continued increases in mortality rates during the post-acute period.

State	Pre-pandemic (2018-2019)	Pandemic (2020-2021)	Post-acute (2022-2024)	% change (pan vs. pre)	% change (post vs. pan)
South Dakota	3.3 (2.4-4.3)	5.2 (4.0-6.5)	4.6 (3.7-5.4)	+56.5%	-12.3%
Oregon	2.6 (2.2-2.9)	4.0 (3.5-4.4)	3.5 (3.1-3.8)	+55.3%	-12.4%
Arkansas	7.8 (7.1-8.5)	11.0 (10.1-11.8)	12.9 (12.1-13.7)	+41.2%	+17.6%
Iowa	3.6 (3.1-4.1)	5.0 (4.5-5.6)	5.9 (5.4-6.4)	+38.9%	+17.5%
Nebraska	2.5 (1.9-3.1)	3.4 (2.8-4.0)	5.7 (5.1-6.4)	+36.5%	+69.2%

Indeed, state-level analyses during the post-acute period (2022-2024) revealed different temporal patterns between the two etiologies. While K70 mortality declined in several states, including Hawaii (-52%) and Alaska (-29.6%), mortality from CLD and cirrhosis not coded as alcohol-related continued to increase in several states. Notably, K73-K74 mortality in Nebraska and Arkansas increased by an additional +69.2% and +17.6%, respectively, relative to the pandemic-period rates.

These state-level findings are descriptive and demonstrate geographic heterogeneity in observed mortality trajectories. Large relative percentage changes should be interpreted cautiously, particularly in jurisdictions with smaller baseline mortality counts or lower baseline mortality rates. The observed differences do not establish causal relationships or the specific mechanisms underlying these geographic patterns.

## Discussion

Principal findings

In this national population-based study of US mortality from 2018 through 2024, ALD and CLD and cirrhosis not coded as alcohol-related demonstrated distinct mortality trajectories across the pre-pandemic, pandemic, and post-acute pandemic periods.

The principal finding was a marked etiologic divergence.

Average annual K70 mortality increased by approximately 32.4% from the pre-pandemic to pandemic period, compared with a 14.3% increase for K73-K74. During the post-acute period, K70 mortality declined by approximately 7% relative to the pandemic-period average, whereas K73-K74 mortality increased by approximately 5.2%.

The YPLL findings further demonstrated that the mortality burden associated with K70 was disproportionately premature. Average annual YPLL increased by 38.8% during the pandemic for K70 compared with 19.5% for K73-K74, and YPLL per death remained substantially higher for K70. Together, these findings demonstrate that pandemic-period changes in liver disease mortality differed across etiologies and were accompanied by differences in premature mortality burden.

Comparison with previous literature

Our findings build on a substantial body of literature demonstrating increasing liver disease mortality before and during the COVID-19 pandemic. Tapper and Parikh demonstrated increasing cirrhosis mortality beginning in 2009, with the greatest relative increases among younger adults and evidence that these increases were driven by ALD [1]. Kim et al. subsequently demonstrated changing etiology-specific mortality trends before the pandemic, including substantial increases in ALD [2]. During the COVID-19 pandemic, Kim et al. demonstrated accelerated CLD and cirrhosis mortality, particularly among younger adults [6]. Their etiology-specific analysis also demonstrated a marked acceleration in ALD mortality during the pandemic [7]. Gao et al. similarly reported substantial pandemic-era changes in liver disease-related mortality, particularly among younger adults and American Indian or Alaska Native populations [8]. Paik et al. extended these observations through 2021 and demonstrated substantial increases in cirrhosis-specific and CLD-related mortality during the pandemic, with pronounced disparities by race and sex [9]. Our findings are consistent with these previous observations but extend them by directly comparing K70 with K73-K74 within the same national population and temporal framework.

Positioning against existing studies

Abboud et al. represent an important methodological comparator because their study evaluated CLD and cirrhosis mortality using national mortality data, although their analytic framework differed from the present study [10]. The present study builds upon this work by explicitly separating the underlying-cause categories represented by K70 and K73-K74 and evaluating their trajectories independently.

Similarly, Pan et al. demonstrated substantial increases in ALD mortality through 2022, while Yu et al. extended ALD mortality analyses through 2024 [13,14]. The present analysis therefore does not seek to replicate the already documented increase in ALD mortality alone. Instead, it evaluates whether the magnitude and temporal direction of mortality changes differed according to whether the underlying cause of death was coded as alcohol-related. The central contribution of this study is therefore the direct etiologic comparison between K70 and K73-K74.

Divergent etiologic trajectories

The observed divergence may be consistent with differences in the pathways through which the pandemic period was associated with liver disease mortality. ALD may have been particularly sensitive to changes in alcohol consumption, social isolation, psychological stress, and access to treatment for alcohol use disorder. National mortality data demonstrated increased alcohol-related mortality during the early pandemic period, and modeling studies projected that increased alcohol consumption could contribute to additional ALD morbidity and mortality [5,15]. However, mortality data cannot establish that increased alcohol consumption caused the observed K70 increase. The persistence of K73-K74 mortality into the post-acute period may be consistent with potential contributions from delayed diagnosis, interruptions in surveillance, reduced specialty care, and disruptions in the management of metabolic and viral liver disease. However, these potential mechanisms were not directly measured in the present study.

Thus, the differing temporal patterns may reflect multiple overlapping factors, but the present ecological analysis cannot determine the relative contribution of specific pandemic-related mechanisms.

Age and sex disparities

The YPLL findings provide evidence that K70 mortality affected a relatively younger population than K73-K74 mortality. This is consistent with previous evidence demonstrating increasing liver disease mortality among younger adults. Tapper and Parikh reported the greatest relative increase in cirrhosis mortality among adults aged 25-34 years, driven by alcohol-related disease [1].

More recent studies have similarly identified younger adults as a population with particularly rapid increases in ALD mortality [8,13,14]. Sex differences were also prominent. Men continued to experience substantially higher absolute K70 mortality. However, women experienced a larger relative increase during the acute pandemic period. This pattern is consistent with recent national studies showing that although men continue to have higher ALD mortality, women have experienced faster proportional increases during periods of rising mortality [13,14]. The findings therefore indicate that absolute sex disparities remain substantial, while women experienced a greater proportional mortality surge during the acute pandemic period.

Race and ethnicity

The racial findings demonstrate substantial etiologic heterogeneity across demographic groups. American Indian or Alaska Native populations experienced the highest absolute K70 mortality rates and the largest relative surge during the acute pandemic period. Conversely, the comparatively stable K73-K74 mortality rates within the American Indian or Alaska Native population demonstrate that the observed temporal changes differed by liver disease category.

Importantly, these observed disparities cannot be attributed to a single behavioral or healthcare-related mechanism based on the present data. Potential contributors may include structural inequities, including historical disparities in healthcare infrastructure, geographical barriers to specialized hepatology and addiction care, and systemic differences in diagnostic ascertainment. Social determinants, healthcare access, historical inequities, behavioral health, geographic isolation, and differences in disease ascertainment may all contribute; however, these factors were not directly measured in the present study. Previous studies have identified particularly high ALD mortality among American Indian or Alaska Native populations and substantial variation among other racial and ethnic groups [8,9,11-14,16].

The present study extends this literature by examining whether these disparities differ according to underlying liver disease category.

Urbanization and geographic disparities

Nonmetropolitan populations experienced higher mortality rates for both etiologies. However, the pandemic-associated relative increase was greater for K70 than K73-K74 in nonmetropolitan populations. This pattern is consistent with recent evidence demonstrating important geographic heterogeneity in ALD mortality and greater late-period increases in nonmetropolitan areas [14].

Urbanization should nevertheless be interpreted as an ecological indicator rather than an individual-level causal factor. The observed rural disparity may be associated with differences in alcohol availability and consumption, access to addiction treatment, hepatology services, transportation, preventive care, socioeconomic conditions, and healthcare infrastructure [17]. These potential mechanisms were not directly evaluated in the present study.

State-level heterogeneity

The marked state-level variation reinforces the importance of geographic context. Several states experienced very large K70 increases during the pandemic followed by partial attenuation, whereas some states experienced continued K73-K74 increases during the post-acute period. This suggests that national averages may obscure important local trajectories.

These different state-level patterns demonstrate geographic heterogeneity in observed mortality changes but do not establish the specific factors responsible for these differences. State-level analyses should therefore be considered descriptive and hypothesis-generating rather than causal. In addition, very large relative percentage changes should be interpreted cautiously, particularly in jurisdictions with smaller baseline mortality counts or lower baseline mortality rates.

Premature mortality and YPLL

YPLL provides an important complementary measure of liver disease mortality. Unlike age-adjusted mortality rates, YPLL places greater weight on deaths occurring at younger ages [18]. This is particularly relevant to ALD because younger adults have experienced some of the fastest increases in alcohol-related mortality [1,8,13,14,18].

In the present analysis, K70 consistently produced substantially greater YPLL per death than K73-K74.

The increase in K70 YPLL during the pandemic was also greater than the corresponding increase in K73-K74 YPLL.

Therefore, the pandemic-era increase in K70 mortality was not simply an increase in the number of deaths; it was associated with a substantial premature mortality burden.

Public health implications

These findings may have several implications for public health and clinical practice. First, the marked pandemic-era escalation in K70 mortality underscores the importance of evidence-based alcohol screening and brief intervention, along with the early detection of ALD and linkage to treatment for alcohol use disorder [19]. Second, the persistent post-acute elevation of K73-K74 mortality highlights the importance of continued CLD surveillance, primary care management, and routine diagnostic follow-up. Third, the observed burden among American Indian or Alaska Native and nonmetropolitan communities underscores the importance of considering geographic and population-specific approaches to prevention and treatment. Finally, while observational mortality data cannot definitively establish a direct causal link to pandemic-era healthcare disruptions, the ongoing elevation of liver disease mortality into the post-acute phase indicates that elevated population-level mortality persisted beyond the acute pandemic period, although the underlying mechanisms cannot be determined from these data.

Strengths

This study possesses several key methodological strengths. First, it utilizes national population-based mortality data covering the entire United States through 2024. Second, it directly contrasts ALD (K70) against CLD and cirrhosis not coded as alcohol-related (K73-K74) using underlying cause-of-death designations. Third, by evaluating three distinct, predefined temporal periods (pre-pandemic, acute pandemic, and post-acute recovery), the analysis captures dynamic multi-year trajectories rather than relying on a simplistic two-period comparison. Fourth, extending surveillance through 2024 provides critical insight into persistent post-acute health impacts. Fifth, the study incorporates comprehensive demographic and geographic stratification, accounting for sex, race, Hispanic ethnicity, degree of urbanization, and state-level variation. Sixth, the integration of YPLL-75 offers a robust quantification of premature mortality burdens. Finally, this approach allows the identification of etiologic differences in mortality trajectories that may be obscured when aggregate CLD and cirrhosis mortality is analyzed as a singular outcome.

Limitations

Several limitations should be considered when interpreting these findings. First, mortality estimates rely on death certificate data, which remain susceptible to diagnostic misclassification or underreporting of liver disease etiology. Second, the K73-K74 category encompasses CLD and cirrhosis not coded as alcohol-related; this designation does not entirely exclude underlying alcohol exposure from disease pathogenesis. Third, given the observational, population-based study design, these data establish temporal associations rather than direct causality regarding pandemic-era disruptions.

Fourth, crude mortality rates were used extensively for subgroup and geographic comparisons. Because populations may differ in age structure, differences in crude mortality rates may partly reflect differences in population age distributions. Although age-adjusted mortality rates were also calculated and standardized to the 2000 US standard population, certain subgroup and geographic analyses relied on crude rates because of CDC WONDER data availability and extraction constraints. Therefore, these comparisons should be interpreted with consideration of potential differences in age structure.

Fifth, CDC WONDER lacks individual-level clinical data, including granular measures of alcohol consumption, alcohol use disorder severity, health insurance status, income, or healthcare utilization. The study also did not directly measure alcohol consumption, access to addiction treatment, healthcare disruption, delayed diagnosis, or interruptions in CLD care. Therefore, explanations involving these factors should be considered plausible hypotheses rather than demonstrated mechanisms.

Sixth, urbanization designations represent county-level ecological metrics and cannot be inferred as individual-level determinants of health. Seventh, the aggregation of seven years of mortality data into three broad periods may obscure year-to-year and within-period variation. The reported percentage changes are descriptive comparisons between prespecified periods and do not constitute formal statistical tests of temporal trends or establish statistically significant changes in mortality trajectories.

Eighth, state-level percentage changes, particularly very large relative increases, should be interpreted cautiously because large percentage changes may occur when baseline mortality counts or rates are relatively small. State-level findings are descriptive and hypothesis-generating and do not establish causal relationships or the specific mechanisms underlying geographic differences.

Ninth, race and ethnicity data reflect administrative classifications that may not fully capture the complex social and structural determinants driving health inequities. Finally, although the study extends through 2024, recent ALD-specific studies have characterized K70 mortality through 2024 [14]. Therefore, the novelty of the present study lies principally in the etiologic comparison between K70 and K73-K74 and the evaluation of divergent trajectories across pandemic-related periods.

An additional limitation is that 2021 intercensal population estimates were used as fixed proxy denominators for certain post-acute urbanization analyses covering 2022-2024 because updated denominators were unavailable or suppressed in CDC WONDER. Although this approach allowed the estimation of crude mortality rates while preserving the available mortality counts, using a fixed 2021 denominator may have introduced additional imprecision or bias into post-acute rate estimates if the underlying population size or geographic distribution changed between 2021 and 2024.

Furthermore, because updated census population denominators for specific urbanization strata (metropolitan vs. nonmetropolitan) for the years 2022-2024 were suppressed or not yet fully integrated into the CDC WONDER extraction framework at the time of our analysis, the 2021 intercensal denominator was utilized as a fixed proxy for the post-acute period. While this approach is mathematically necessary to estimate crude rates without discarding the highly valuable, verified absolute mortality counts (the numerators) of the post-acute period, it operates under the assumption of population stability within those geographic strata between 2021 and 2024.

## Conclusions

In this national population-based analysis of US mortality from 2018 through 2024, ALD and CLD and cirrhosis not coded as alcohol-related demonstrated divergent mortality trajectories across the pre-pandemic, pandemic, and post-acute pandemic periods. K70 mortality exhibited a pronounced increase during the acute pandemic period followed by partial attenuation, whereas K73-K74 mortality increased more gradually and remained elevated during the post-acute period.

The substantially greater YPLL burden associated with K70 indicates a disproportionately high burden of premature mortality associated with ALD. The marked heterogeneity by sex, race, urbanization, and state further demonstrates that these observed mortality patterns varied across population subgroups.

These findings demonstrate temporal and etiologic differences in liver disease mortality but do not establish that specific pandemic-related mechanisms, including changes in alcohol consumption or healthcare disruption, caused the observed patterns. The findings may nevertheless help identify populations and disease categories that warrant further investigation and targeted prevention, treatment, and surveillance efforts.
